# Effects of Drought, Pest Pressure and Light Availability on Seedling Establishment and Growth: Their Role for Distribution of Tree Species across a Tropical Rainfall Gradient

**DOI:** 10.1371/journal.pone.0143955

**Published:** 2015-11-30

**Authors:** Julian Gaviria, Bettina M. J. Engelbrecht

**Affiliations:** 1 Department of Plant Ecology, Bayreuth Center of Ecology and Environmental Research (BayCEER), University of Bayreuth, Bayreuth, Germany; 2 Smithsonian Tropical Research Institute, Balboa, Ancón, Panama; Wuhan Botanical Garden,CAS, CHINA

## Abstract

Tree species distributions associated with rainfall are among the most prominent patterns in tropical forests. Understanding the mechanisms shaping these patterns is important to project impacts of global climate change on tree distributions and diversity in the tropics. Beside direct effects of water availability, additional factors co-varying with rainfall have been hypothesized to play an important role, including pest pressure and light availability. While low water availability is expected to exclude drought-intolerant wet forest species from drier forests (physiological tolerance hypothesis), high pest pressure or low light availability are hypothesized to exclude dry forest species from wetter forests (pest pressure gradient and light availability hypothesis, respectively). To test these hypotheses at the seed-to-seedling transition, the potentially most critical stage for species discrimination, we conducted a reciprocal transplant experiment combined with a pest exclosure treatment at a wet and a dry forest site in Panama with seeds of 26 species with contrasting origin. Establishment success after one year did not reflect species distribution patterns. However, in the wet forest, wet origin species had a home advantage over dry forest species through higher growth rates. At the same time, drought limited survival of wet origin species in the dry forest, supporting the physiological tolerance hypothesis. Together these processes sort species over longer time frames, and exclude species outside their respective home range. Although we found pronounced effects of pests and some effects of light availability on the seedlings, they did not corroborate the pest pressure nor light availability hypotheses at the seed-to-seedling transition. Our results underline that changes in water availability due to climate change will have direct consequences on tree regeneration and distributions along tropical rainfall gradients, while indirect effects of light and pests are less important.

## Introduction

Tropical forests are among the most diverse communities worldwide. Changes of rainfall and soil moisture with global climate change will potentially have dire consequences for tropical forests; however, the uncertainty of projections remains high [1]. One of the most prominent patterns in tropical forests is an increase of tree species richness with rainfall and a decrease with dry season intensity (e.g. [2]). At the same time, tree distribution and forest composition are strongly related to rainfall, and species turn-over is high across tropical rainfall gradients [3–6]. Understanding the mechanisms underlying tree distribution patterns, community composition and diversity across rainfall gradients is necessary to improve projections of the effects of global change on tropical forests and to optimize management, conservation and restoration strategies.

Several factors have been hypothesized to shape tree distribution patterns across rainfall gradients, including direct effects of water availability. According to the physiological tolerance hypothesis [7], drought-intolerant species are excluded from dry forests, thus leading to differences in species composition and species numbers among dry and wet forests. The direct role of drought tolerance, i.e. the ability to withstand periods of low water availability, in limiting wet forest species from occurring in forests with a pronounced dry season is supported by experimental studies [8–10]. However, at the same time many dry forest species do not occur in wet sites [8, 11, 12]. The physiological tolerance hypothesis thus fails to explain a large part of variation of tree distribution [8] and the high species turnover observed across tropical rainfall gradients [3]. Other environmental factors that co-vary with rainfall have been hypothesized to indirectly influence tree species distributions. These include increases of insect herbivore and pathogen pressure (summarized as pest pressure) and decreases of light availability with rainfall [2, 10, 13–18].

Herbivores and pathogens have long been hypothesized to influence species distributions and diversity along tropical rainfall gradients [16, 17]. According to the pest pressure gradient hypothesis [13], species originating from dry forests with low herbivore pressure are less defended and therefore excluded from wet forests with high herbivore pressure. Despite its potential importance for explaining community compositions in tropical forest, empirical support for this hypothesis remains scarce. Evidence for changes of pest pressure with rainfall or moisture remains contradictory, and no differences of herbivore nor pathogen damage between species origins have been found in reciprocal transplant experiments at the seedlings stage, indicating that defenses did not differ between species of dry, seasonal and wet, aseasonal forests [13, 14, 19]. Thus, the relevance of the pest pressure hypothesis for explaining species distributions remains to be shown.

Light availability has been hypothesized to influence species distributions along rainfall gradients, by excluding light-demanding dry origin species from wet forests with low understory light levels [15, 18]. Higher light requirements of dry forest species have been hypothesized as a consequence of a trade-off between shade and drought tolerance [18, 20], based mainly on a trade-off between biomass allocation to roots, which would confer drought tolerance, and allocation to leaves, which confers shade tolerance. However, there is no conclusive support for a trade-off between drought and shade tolerance in tropical forest plants [8, 21–23], as traits conferring drought or shade tolerance are complex, not necessarily related and can be uncoupled. Higher light requirements of dry forest species have also been hypothesized due to their evolution in higher light environments in dry forests [15, 20]. Although lower light conditions in wetter forests have long been assumed [20, 24], few studies have directly compared light availability along rainfall gradients [15, 25]. The results do not support that there is a general pattern [25]. Instead, nutrients and species composition additionally strongly influence forest structure and understory light availability [26]. Thus, the role of light in shaping species distributions across rainfall gradients also remains unclear.

Apart from environmental factors, intrinsic trade-offs between stress tolerance and growth rate [20, 27], may also lead to exclusion of drought-tolerant dry origin species from wet forests. Adaptations to stressful, resource-limited environments have been hypothesized to be coupled with intrinsically low growth rates, based on biomass investment into either roots, which confer higher drought (stress) tolerance or into leaves, which allows for higher growth rates. Other traits that confer drought tolerance, like high wood density, small vessel diameter or high non-structural carbohydrate concentrations, are also associated with low growth rates [28, 29]. Thus, drought-tolerant dry origin species should have intrinsically lower growth rates, which put them at a disadvantage when water is not limiting as in wet forests. Under such conditions, they may thus be outcompeted by drought-intolerant, fast-growing wet origin species. However, at the level of whole-plant performance, evidence for a drought tolerance-growth trade-off and its role for species distributions across rainfall gradients remains scarce and contradictory (e.g. [30–32]).

Plants responses to drought, pest pressure and light availability differ among life stages. Early life stages, especially seedling emergence, are considered vulnerable to abiotic and biotic stressors [19, 33, 34], and may thus be critical in shaping species distributions. Plant defenses often increase with ontogeny [35], and the same absolute amount of leaf damage should have larger impact on small seedlings compared to bigger, older plants, thus rendering initial life stages especially vulnerable to pests. Experimental studies on factors shaping tree distributions across rainfall gradients have so far mainly focused on established seedlings ([8, 9, 13–15, 36], but see [19]). In our study we therefore specifically focused on the role of seed-to-seedling transition and first-year establishment for distribution patterns.

**Fig 1:**
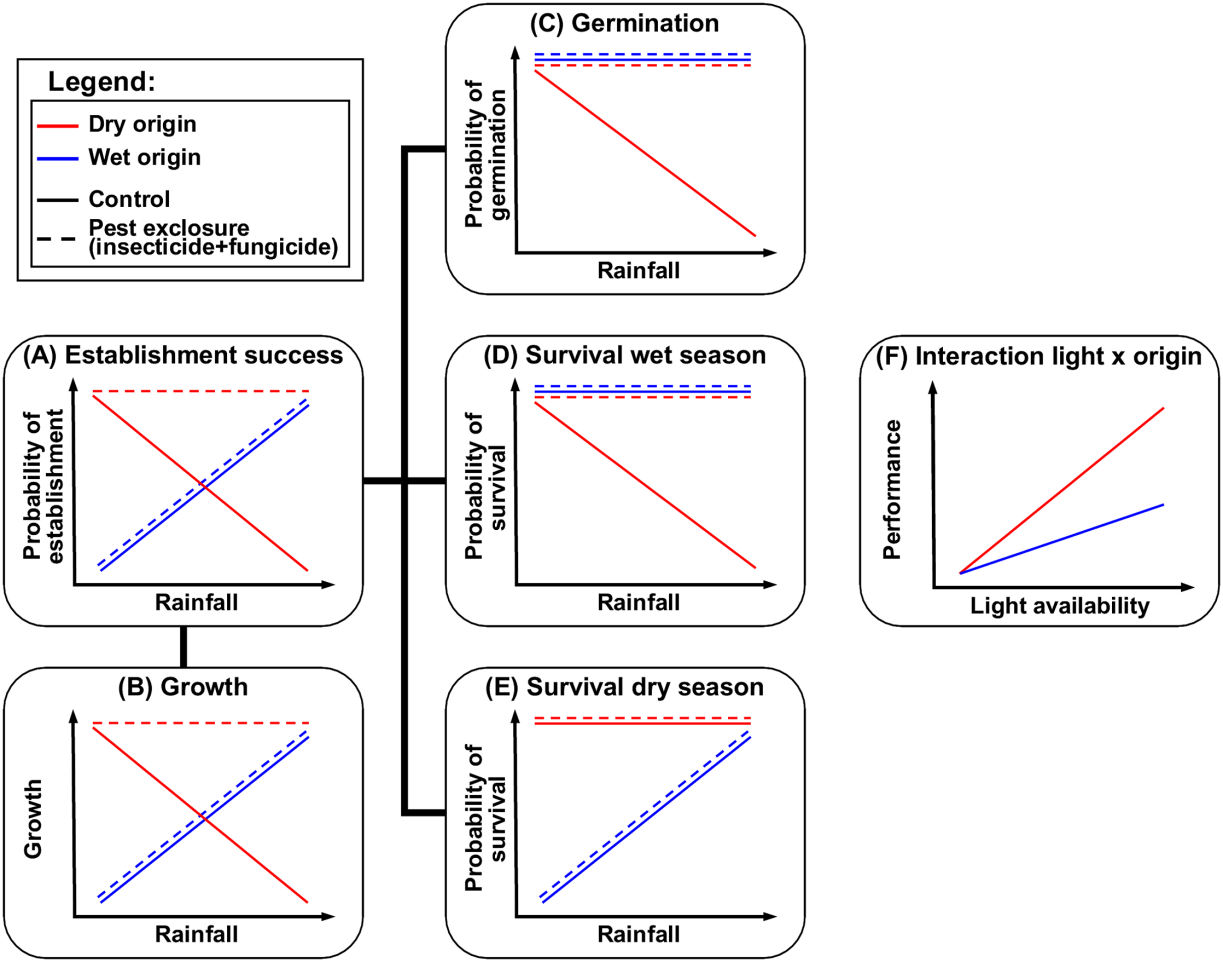
Schematic representation of the specific hypotheses for the effects of drought, light and pests on seedling performance. For overall probability of establishment success (A) and growth (B), we hypothesized that species perform better in their respective home range outcompeting foreign species, with drought limiting wet origin species in the dry site, and pest damage limiting dry origin species in the wet site. Consequently, we expected that the pest exclosure enhances performance only for (poorly defended) dry origin species in the wet site (with high pest pressure) (i.e. three-way interaction between treatment (exclosure/control), origin (dry/wet) and site (dry/wet)). For germination (C) and survival in the wet season (D), when water availability and pest pressure are assumed to be high, we expected that wet origin species have higher survival than dry origin species in the wet site, under control conditions (site x origin interaction). We expected that (poorly defended) dry origin species are limited by pest pressure in the wet site, indicated by higher performance when pest pressure is alleviated through pest exclosure (three-way site x origin x treatment interaction). In contrast, (well defended) wet origin species exhibit no differences in germination/survival between sites, independent of the pest exclosure. We expected dry season survival (E) of (drought sensitive) wet origin species to be lower than survival of dry origin species in dry sites (significant site x origin interaction). Because pest pressure is assumed to be lower in the dry season, we expected no increase in survival with pesticide treatment for any combination of site and origin (no three-way interaction). With increasing light availability (F) we expected a stronger increase in all performance parameters for dry origin species (light x origin interaction), reflecting their higher light requirements.

The aim of this study was to test how the combined effects of drought, pests and light availability affect early seedling performance of tree species with contrasting origins (dry vs. wet), and how these differences in seedling performance influence species distribution patterns. We hypothesized that species have a performance advantage within their respective home (native) range compared to foreign (alien) species, resulting in exclusion of the foreign species. We expected that drought limits performance of wet forest species in drier sites (physiological tolerance hypothesis), and that pests and/or light availability limits the performance of dry forest species in wetter sites (pest pressure and light availability hypothesis, respectively). To test these hypotheses, we conducted a reciprocal transplant experiment along a rainfall gradient in Panama, with species with contrasting origins. Pests were excluded for half of the seeds, and light and soil moisture conditions were monitored during one year, including a dry and a wet season. Specific expectations for plant performance in the experiment are depicted in Fig 1.

## Materials and Methods

### Study sites

The study was conducted at the Isthmus of Panama, which exhibits a pronounced rainfall gradient from 1600 mm/year at the Pacific Coast to over 3000 mm/year at the Atlantic coast across a distance of only 65 km [4, 8]. The length of the dry season, which typically starts in January and ends in May, correlates negatively with annual rainfall [8]. Mean annual temperature is 25°C with little variation across the gradient or throughout the year.

The experiment was conducted in two forests about 50 km apart: a drier semi- deciduous forest located in the national park Camino de Cruces (9° 2'N, 79°35'W, 2000 mm annual rainfall; modeled based on BIOCLIM data, [37]), and a wetter evergreen lowland forest in the national park San Lorenzo (9°16'N, 79°58'W, 3200 mm annual rainfall). Both sites are located in the Tropical Moist Forest Life Zone [38, 39]. However, rainfall and moisture regime, as well as species composition vary greatly. Dry season length is approximately 150 and 120 days, and rainfall in the driest quarter of the year 530 mm and 800 mm [8, 38], respectively. Soil water potentials in the upper soil layer of the dry site reach values well below -2 MPa in the dry season, but remain high throughout the year in the wet site (Engelbrecht, unpublished data). Both forest sites were mature secondary forest located on sedimentary bedrock. Only about 10% of the species in the areas overlap [3, 38]. In the following we refer to these sites as “dry” and “wet”, respectively. Permits for working in the national parks were granted by the “Autoridad Nacional del Ambiente (ANAM).

### Experimental design

At each forest site, 60 paired plots (90 cm x 90 cm) were established, with each pair including a pesticide treatment (fungal pathogen and insect herbivore exclosure, see below) and a control plot (2 sites x 2 treatments x 30 plots). Seeds of 15 species with “wet origin” and 11 species with “dry origin” (S1 Table, for definitions see below) were sown into each plot, with one seed of each species in each plot. Germination, seedling survival in the dry and the wet season, and growth were followed over one year.

### Seedling plots

The plots were set-up in the forest understory avoiding any gaps, with pairs separated at least 70 m from each other, spanning an overall area of about 300 ha in San Lorenzo (wet site) and 150 ha in Cruces (dry site). The control and exclosure plots were separated by at least 2 m. Where relevant, the control plots were set-up uphill from the exclosure plots to prevent runoff from the treatment to the control (three times in San Lorenzo and two times in Cruces). To allow access of insect herbivores but prevent seed or seedling removal by rodents or other mammals, which were not the focus of this study, all plots were caged with 2.5 x 2.5 cm wire mesh.

### Study species and plant material

Study species were selected to include common species with strong association to the dry or the wet side of the isthmus. We focused on shade-tolerant species, since they represent about 80% of the species in these forests [40, 41]. Species with small seeds (< 0.5 cm length) were excluded to facilitate their manipulation and localization in the field. Potential study species were selected based on their occurrence in 50 1 ha plots spanning the rainfall gradient [42] and/or their abundance in a wet and a dry forest plot (Sherman, 6 ha and Cocoli, 4 ha, respectively, see [42]). Species with predominantly wet Caribbean side occurrence, that did not occur on the dry Pacific side of the Isthmus, or that had at least double the abundance in the wet than the dry side plots were classified as “wet origin species”, whereas species occurring predominantly on the dry Pacific side, that did not occur on the wet Caribbean side of the Isthmus, or that had at least double the abundance in the dry than the wet side plots were classified as “dry origin species” (S1 Table).

Seeds were collected in mature secondary forests across the Isthmus within their respective natural home range in the national parks San Lorenzo, Soberania, Chagres and Camino de Cruces during the dry season and beginning of the wet season 2012 (March to mid-May). Ripe seeds were collected from a minimum of three mother trees per species by directly harvesting from the tree, or from freshly fallen fruits. Damaged seeds were removed after visual inspection for damage by predators or pathogens. Final selection of the study species was based on the availability of enough undamaged seeds, resulting in 15 “wet” and 11 “dry” origin species.

### Pest exclosure treatment

To exclude fungal pathogens and insect herbivores (summarized as pests) a combination of a fungicide and an insecticide (summarized as pesticides) was applied monthly to the treatment plots. Actara (active ingredient: Thiamethoxam), a systemic broad-spectrum insecticide, and Diligent (active ingredients: Methalaxyl and Chlorothalonil), a systemic broad-spectrum fungicide with protectant properties effective against true fungi as well as oomycetes were used. According to the specification of the manufacturer, Actara was used in a solution of 0.5 g/l water, and Diligent in a solution of 5 g/l. Each exclosure plot was sprayed with a mixture of 40 ml of the insecticide and 40 ml of the fungicide solution. The control plots were sprayed with the same amount of rainwater, to ensure that results were not biased by additional water availability in the treatment. Studies using similar pesticide treatments have discarded negative influences on non-target organisms, including the plants themselves [43, 44]. Seeds in the exclosure plots were additionally pre-treated with the broad-spectrum insecticide Brigadier (active ingredient: Bifenthrin), and the fungicide Diligent (see above) to avoid seed predation. The insecticide was used undiluted with 50 ml/kg seeds. The seeds were briefly soaked in both solutions. Seeds of the control plots were soaked in rainwater.

### Seed sowing

120 seeds per species (2 sites x 2 treatments x 30 plots, with one seed per species per plot, totaling 3600 seeds) were sown at the end of the dry season/beginning of the wet season (starting in March 2012). To ensure high germination rates in the typically recalcitrant seeds and to mimic natural seeding periods, seeds were sown as soon as possible after collection (maximum two days later), and distributed evenly between exclosure and control plot and wet and dry site (i.e. not all seeds of one species were planted at the same time). Seeds were planted on the mineral soil under the leaf litter, in a 15 x 15 cm grid, with species assigned randomly to the positions. Leaf litter was disturbed as little as possible to ensure natural microhabitat conditions in the plots. To prevent washing away and to facilitate relocation, seeds were fixed to the ground with wooden toothpicks and positions marked.

### Seed germination, survival and growth

Seed germination and seedling survival were monitored between March 2012 and April 2013, i.e. during the transition between initial dry and wet season, a wet season and a second dry season. Rainfall during the study period did not differ substantially from the long-term average, except for almost the double amount of rainfall in November and December 2012 [45]. During the first 3.5 months, the time of highest germination, censuses were conducted biweekly to ensure that all germinating seeds were recorded; radicle emergence was counted as germination. Thereafter, censuses were conducted at monthly intervals for seedling survival, based on aboveground living biomass, and for occasional further germination. Overall growth was assessed at the last census based on seedling height, measured from the ground to the highest meristem.

From the census data we quantified six performance parameters: (1) *overall establishment success* (proportion of remaining seedlings at the end of the experiment relative to the original number of seeds sown; covers the period from March 2012 to April 2013); (2) *overall growth* (height of the seedlings at the end of the experiment in April 2013); (3) *germination* (proportion of seeds that germinated until the end of the experiment, relative to the original number of seeds sown); (4) *survival during the wet season* (proportion of seedlings that survived until December 2012, relative to the number of germinated seeds); and (5) *survival during the dry season* (proportion of seedlings that survived until April 2013, relative to the number of seedlings present at the start of the dry season).

### Soil moisture and light

**Fig 2:**
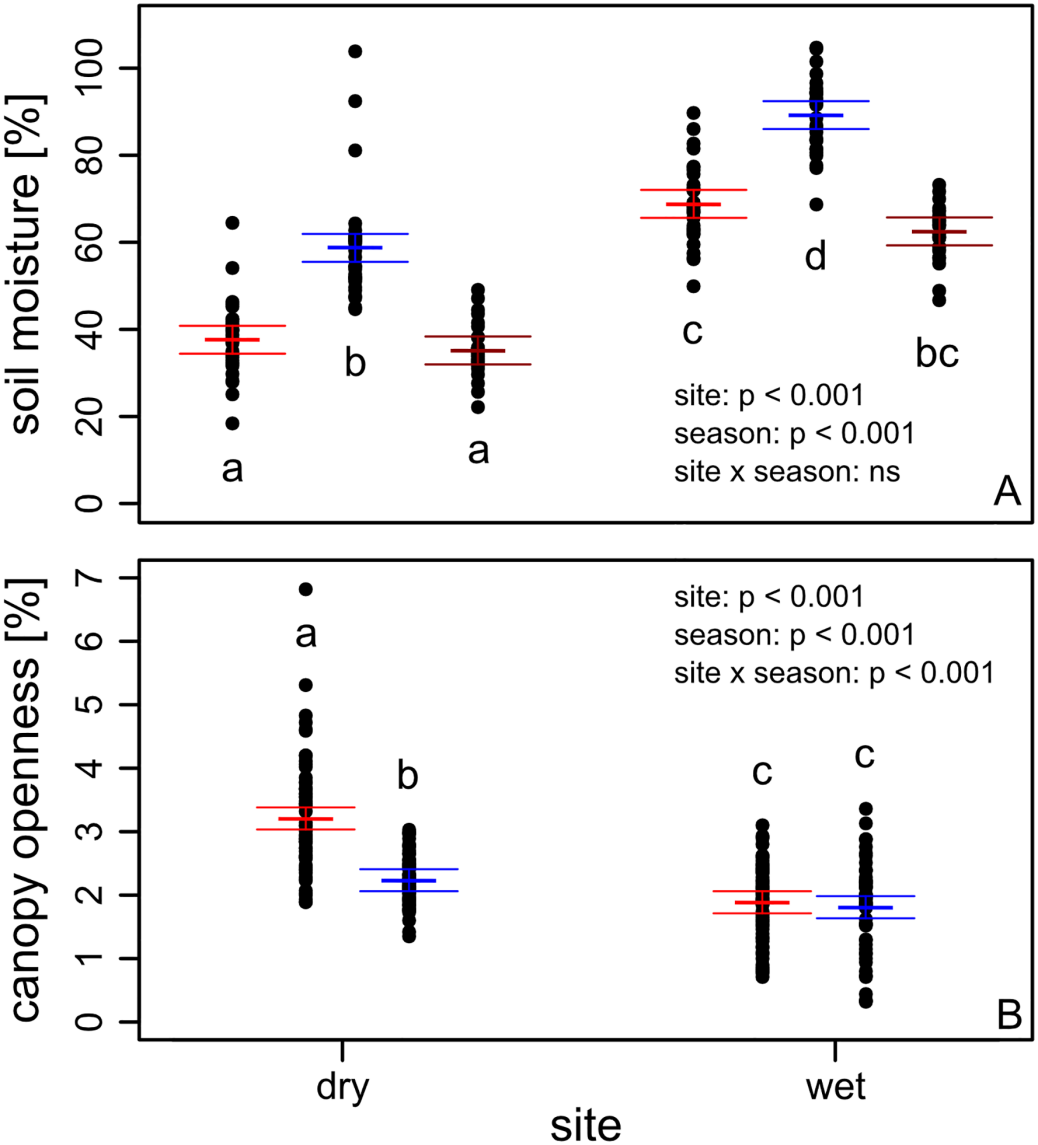
Gravimetric soil moisture (A) and canopy openness (B) in the dry and wet site across seasons. Colors indicate the end of the dry season 2012 (red), wet season 2012 (blue) and dry season 2013 (dark red). Included are results of an ANOVA for effects of site, season and site x season interactions. Different letters represent significant differences at the 0.05 level in a Tukey post-hoc test. Presented are means (thick horizontal lines), 95% CI (thin lines), and raw data (points).

We recorded gravimetric soil moisture at each census, and averaged over the dry and wet seasons, respectively. For light availability, canopy openness was assessed once during the dry and wet season, respectively (Fig 2).

For soil moisture, three random soil cores 15 cm deep were taken within 1 m from each plot pair; fresh and dry weight (after drying to constant weight at 105°C) were determined and percent gravimetric water content was calculated based on dry weight. We assessed canopy openness (percent open sky) from hemispherical photographs taken 1 m over each plot during the dry (April 2012) and during the wet season (October 2012), using a Nikon Coolpix P5000 camera with a Fisheye Converter FC-E8. Photographs were analyzed with the program Gap Light Analyzer v2.

Gravimetric soil moisture and canopy openness varied significantly across sites and between seasons: gravimetric moisture was lower in the dry than in the wet forest and lower in the dry than in the wet season (Fig 2a). Conversely, canopy openness was higher in the dry than in the wet forest, and higher in the dry than in the wet season (Fig 2b).

### Statistical analyses

Our main aim was to assess the effect of site (as a categorization of moisture, see below), species origin, pest exclosure and light on probabilities of germination and survival, as well as on growth.

We initially tested for correlations between the explanatory variables (S2 Table). Soil moisture correlated strongly between seasons as well as with the factor site (r ≥ 0.8, see S2 Table). Therefore, soil moisture and site could not be maintained together in the same model. Models treating soil moisture as continuous variable and models with the factor site (wet/dry, see below) yielded qualitatively the same results. We present results of the models with the factor site, because we were interested in responses to the large-scale rainfall gradient rather than small-scale responses within sites, and because using the factor site better reflected the experimental setup with a separate “dry” and “wet” site.

The performance parameters we analyzed were: (1) *establishment success*, (2) *growth*, (3) *germination*, (4) *survival during the wet season* and (5) *survival during the dry season* (Table 1). To account for heteroscedasticity and non-normality of the residuals, growth data was log-transformed using the natural logarithm.

**Table 1:** Effects of site, species origin, pest exclosure and light on performance parameters of tropical tree seedlings.

	Establishment success	Growth	Germination	Survival wet season	Survival dry season
*single term effects*					
site	ns	**<0.001** (-)	<0.1 (+)	ns	ns
origin	ns	**<0.05** (+)	ns	ns	**<0.05** (-)
treatment	**<0.001** (+)	ns	**<0.001** (+)	**<0.05** (+)	**<0.001** (+)
canopy openness	ns	ns	ns	**<0.05** (+)	ns
*interactions*					
site x origin	ns	**<0.01**	ns	ns	**<0.05**
site x treatment	**<0.01**	ns	**<0.01**	ns	ns
origin x treatment	ns	ns	ns	ns	ns
origin x canopy openness	<0.1	ns	ns	ns	ns
site x origin x treatment	**<0.01**	ns	<0.1	<0.1	ns

Summary of the results of the Generalized Linear Mixed Effects Models (GLMM) and Linear Mixed Effects Model (LMM, for growth) for the six performance parameters. Significant relations are in bold. Detailed results are given in S3 Table.

(+) / (-): positive or negative effect of pest exclosure, wet site, wet origin or high light on performance parameters. These are only given for single-term results, not for the interactions.

We initially assessed species effects on performance by fitting a separate model for each performance parameter (Generalized linear mixed effects with binomial distribution (GLMM), or linear mixed effects (LMM) for growth, see S1 Fig). Species was used as fixed effect factor in each model. Random intercepts were plot-pairs and plots, with plot nested in plot-pairs.

To assess the effects of site, origin, treatment and light availability on performance, one model per performance parameter was fitted (GLMM or LMM, respectively). For every model, fixed effect factors were site (dry/wet), origin of the species (dry/wet), treatment (pest exclosure/control) and the average light availability (canopy openness in %) for the period analyzed (dry season, wet season, annual mean of dry and wet season, respectively). We also included the triple interaction term site x origin x treatment (which includes the pairwise interactions site x origin, site x treatment and origin x treatment), and the interaction term origin x light availability (Table 1). Random intercepts were species, plot-pair and plot. We nested species in plots and plots in plot-pairs. Single term deletion of non-significant terms was used for model selection. We removed sequentially first all interaction terms and then all explanatory variables that led to a model with a lower Akaike Information Criteria (AIC).

To test our specific expectations (Fig 1), we conducted eight planned comparisons using least squares means [46] with Tukey correction as post-hoc tests (Table 2). To assess if seed germination and seedling survival varied across sites or with species origin under the *natural condition* of the habitat, we assessed under control conditions the effect of origin of the species within the dry and the wet site, respectively, and the effect of site on wet origin and dry origin species, respectively (i.e. four contrasts, Table 2a and 2b). To assess to what extent germination and survival were affected by pests, we assessed the pest exclosure effect in each site (wet/dry) and in species with different origin (wet/dry, i.e. four contrasts, Table 2c and 2d). Tukey post-hoc comparisons, means and standard errors in tables and figures are from the least squares table [46].

**Table 2:** Planned comparisons of (A) effects of origins and sites under natural (control) conditions, and (B) effects of pest exclosure on performance parameters.

	A. Effects of site and species origin under natural conditions				B. Effects of pest exclosure within sites and origins			
	a. Effect of origin within sites		b. Effect of site within origin		c. Exclosure effect within dry site		d. Exclosure effect within wet site	
Performance parameter	Dry site	Wet site	Dry origin	Wet origin	Dry origin	Wet origin	Dry origin	Wet origin
Establishment success	ns	ns	ns	ns	**<0.001**	**<0.001**	ns	**<0.001**
Growth	**0.02**	**<0.001**	**<0.001**	ns	ns	ns	ns	ns
Germination	ns	ns	0.06	ns	**<0.001**	ns	ns	ns
Survival wet season	ns	ns	ns	0.05	**0.02**	**0.001**	ns	**<0.001**
Survival dry season	**0.02**	ns	ns	**0.04**	0.09	**0.03**	ns	**<0.001**

(a) Effects of species origin within the dry and the wet site, and (b) effects of site on species with dry and wet origin under control conditions. Effects of exclosure (c) within the dry site on dry origin and wet origin species, and (d) within the wet site, on dry and wet origin species. Post-hoc analyses are based on least squares means contrasts [46] with Tukey correction. Significant contrasts are in bold.

All statistical tests were done using R 3.0.2 [47] with the packages lme4 1.0.5 [48], lsmeans 2.00–5 [46] and LMERConvenienceFunctions 2.5 [49].

## Results

Species differed significantly in overall establishment success, as well as in germination, dry and wet season survival, and growth (all p < 0.001, S1 Fig). Pest exclosure (i.e. pesticide treatment) had an overall significant positive effect on all performance parameters except growth (Table 1), underlining the importance of herbivores and pathogens in limiting seed germination and seedling survival in tropical forests. Light availability (i.e. canopy openness) only had a positive effect on wet season survival (Table 1). Several performance parameters were affected by site and origin or by interactions between site, origin, treatment and light, but these effects differed among performance parameters (Table 1).

**Fig 3:**
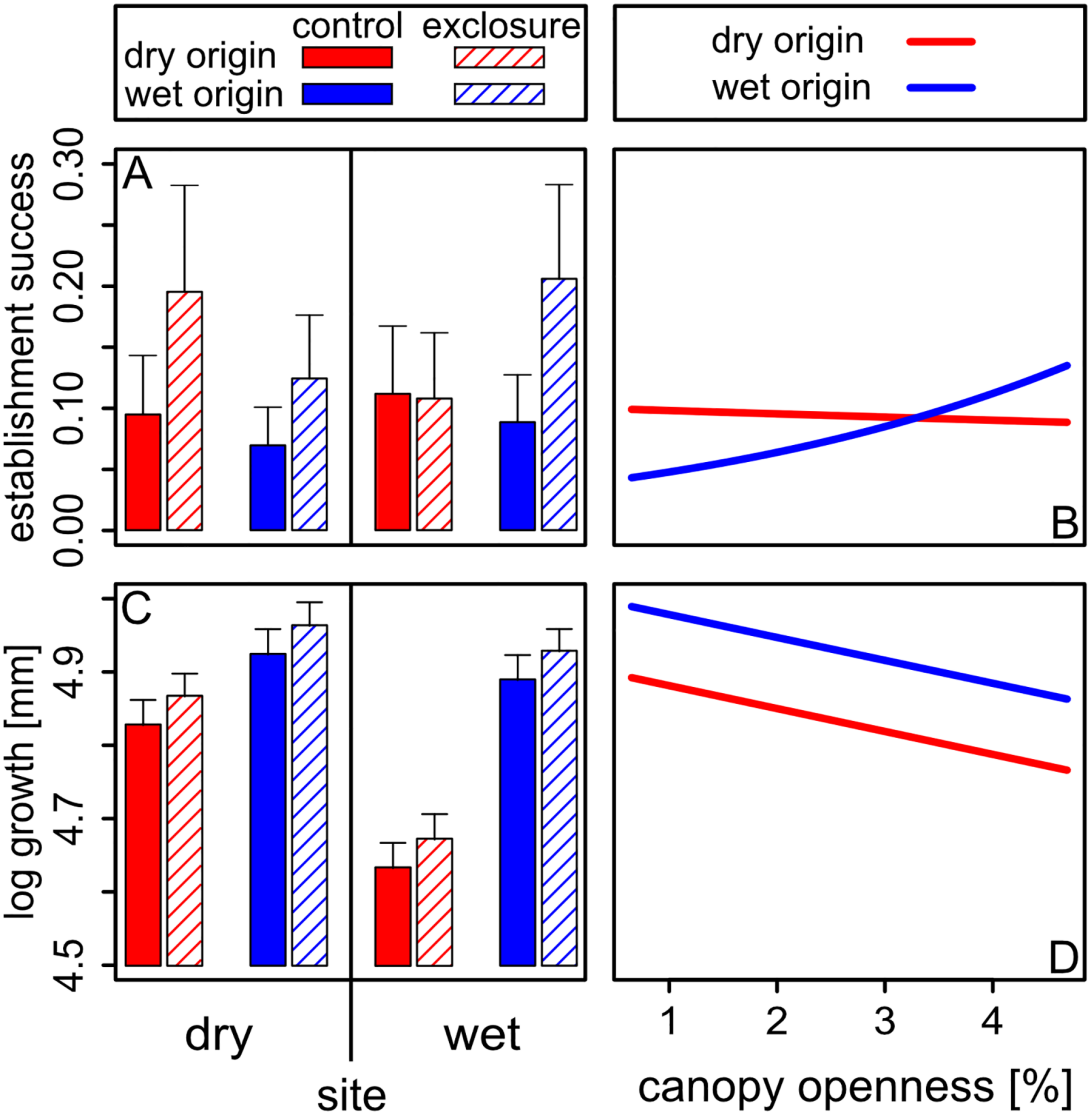
Overall probability of establishment success (A and B) and growth (C and D) at the end of the experiment after one year, as affected by moisture (dry vs. wet site), origin (dry vs. wet), pest exposure (control vs. exclosure) and light availability (canopy openness). Panels A and B show means and standard errors from the least squares means table [46]. For canopy openness (B and D), results of exclosure and control seeds and seedlings were pooled, since we did not expect light availability to influence the effect of the exclosure treatment. For overall analyses see Table 1, for planned contrasts (post-hoc-tests) see Table 2.

**Fig 4:**
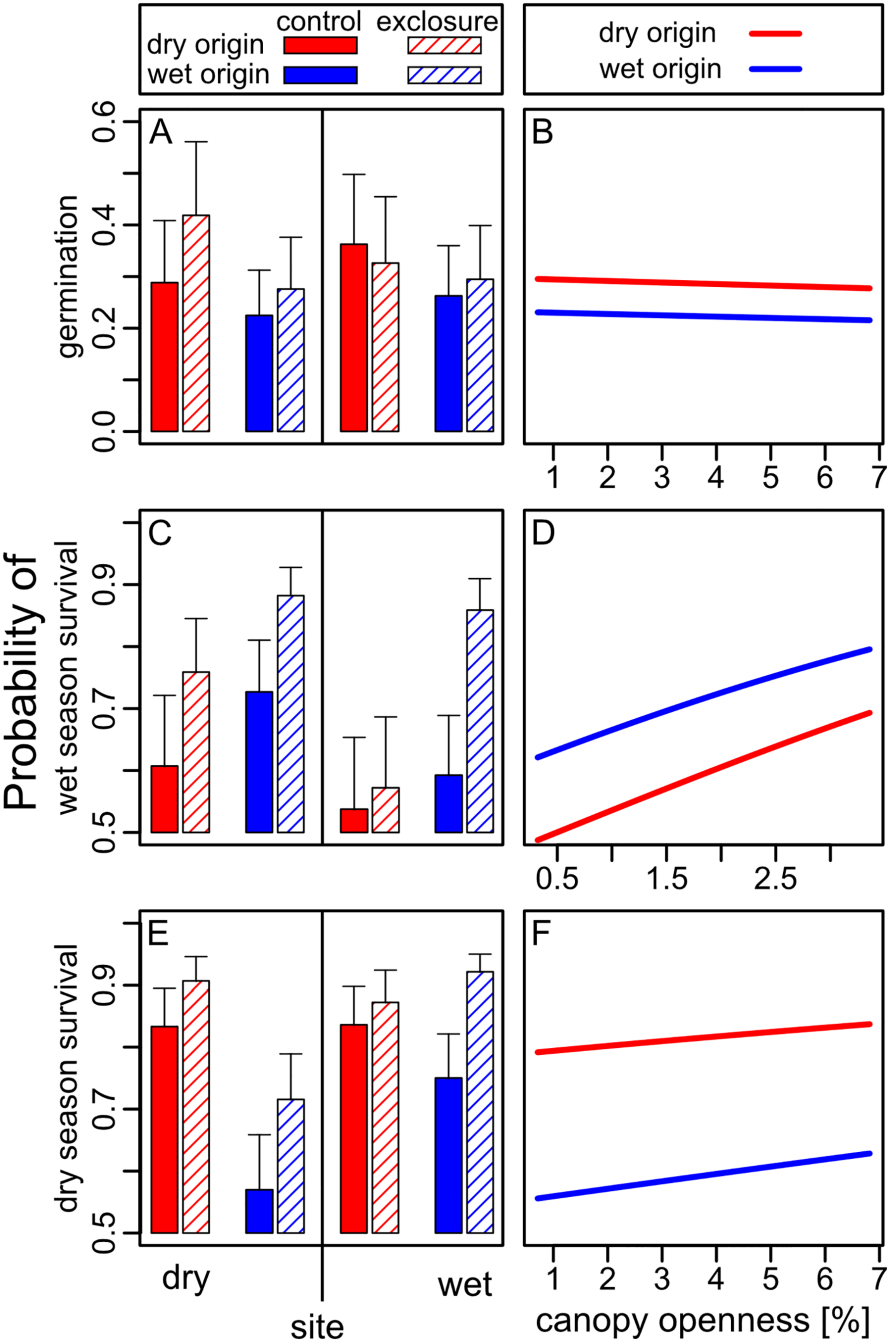
Probability of seed germination (A and B), wet season seedling survival (C and D) and dry season survival (E and F) for species with dry or wet origin as affected by moisture (dry vs. wet site), herbivore exposure (control vs. exclosure) and light availability (canopy openness). Panels A, C and E show means and standard errors from the least squares means table [46]. For canopy openness, results of exclosure and control seeds and seedlings were pooled (see also Fig 3). For overall analyses see Table 1, for planned contrasts (post-hoc-tests) see Table 2.

Below we first present the results for overall seedling establishment and growth during the study (Fig 3). The establishment success after one year is the cumulative result of germination and survival patterns, which are presented separately (Fig 4), and integrates processes in the wet and the dry season over the course of the experiment (see also Fig 1). To test our main hypotheses, we first focus on species performance under natural pest pressure (i.e. controls) to compare the performance of home vs. foreign species (i.e. origins) within sites and across sites (planned comparisons in Table 2a and 2b). Then we focus on the effects of pest exclusion within sites and across origins (planned comparisons in Table 2c and 2d). Finally, we depict the effects of light. Full results of the three-way interactions, as well as of pairwise interactions and individual factors are summarized in Table 1, and details are given in S3 Table.

### Seedling establishment

At the end of the experiment after one year, 22.2% of the seedlings out of all seeds that were sown survived (799 out of 3600). We expected species to have a disadvantage outside their home range exhibiting lower overall establishment success, with dry origin species being limited by pest pressure and/or low light availability in the wet site, and wet origin species being limited by drought in the dry site. Contrary to our expectation, for control seedlings there were no significant differences in the establishment success of the two origins within sites (Table 2a, Fig 3a), nor across sites (Table 2b, Fig 3a). Thus, under natural conditions, dry origin and wet origin species did not differ in their probability to establish in both sites. Pest exclosure significantly enhanced seedling establishment, and the strength of the effect depended on site and origin (significant treatment x site x origin interaction, Table 1, Fig 3a): the pest exclosure enhanced establishment success of wet origin species on both sites (Table 2c and 2d), but of dry origin species only in the dry site (Table 2c). Thus, contrary to our expectations, pest exclosure had no positive effect on dry origin species in the wet site (Table 2d). Light had no overall significant effect on the establishment success over the whole experimental period. There was a marginally significant origin x light interaction; however, opposite to our expectations, wet origin species profited more from higher light availability than dry origin species (Table 1, Fig 3b). None of the other performance parameters exhibited a significant light x origin interaction (Table 1).

### Growth

On average, after one year species had grown to a height of 14.1 cm (3.5–62.5 cm). We found a significant site x origin interaction (Table 1): Dry origin species grew significantly less than wet origin species in the wet site (Fig 3c). Even in the dry site dry origin species grew less than wet origin species, although the difference was less pronounced than in the wet site (Table 2a, Fig 3c). Dry origin species grew less in the wet site than in their dry home range, while wet origin species showed no difference in growth performance across sites (Table 2b, Fig 3c). Independent of species origin, overall growth was lower in the wet site compared to the dry site (Table 1). Dry origin species had an overall lower growth than wet origin species independent of site (Table 1). Pest exclosure did not affect growth, neither alone nor in interaction with origin nor site, indicating that pests did not limit growth in our experiment (Table 1, Table 2c and 2d). Growth was also not affected by the variation of light availability encountered in this study (Table 1, Fig 3d).

### Germination

Out of the seeds sown in the experiment, 38% germinated (1384 of 3600). Germination ranged from 0% to 94% among species (S1 Fig). Within sites, no differences were found between origins under natural conditions (Table 2a, Fig 4a). Contrary to our expectations, dry origin species showed a marginally significant trend (p = 0.06) to germinate better in the wet than in the dry site under natural control conditions, while wet origin species did not show any differences between sites (Table 2b). As expected, pest exclosure benefited dry origin species more than wet origin species, but opposite to our expectations, only in their home range (Table 2c and 2d, Fig 4a). Wet origin species showed no higher germination rates when pests were excluded, neither in the dry nor in the wet site (Table 2c and 2d). Light availability did not affect germination, neither for dry nor for wet origin species (Table 1, Fig 4b).

### Seedling survival during the wet season

In the wet season, 72% of the germinated seeds survived (994 of 1384). Although the interaction site x origin x treatment was marginally significant (Table 1), trends did not conform to our expectations (Fig 4c). Wet origin species did not show higher survival rates than the dry origin species in any of the sites (Table 2a, Fig 4c), although we had expected them to show lower mortality during the wet season than dry origin species. Also opposite to our expectations, there was a trend for wet origin species to perform better in the dry site, while dry origin species showed no difference between sites (Table 2b). Pest exclosure benefited wet origin species both in the dry and the wet site, and dry origin species only in the dry site (Table 2c and 2d). Wet season survival of the seedlings increased with increasing light availability, but there were no differences between the two origins (Table 1, Fig 4d).

### Seedling survival during the dry season

The subsequent dry season was survived by 80% of the seedlings (799 of 994). There was a significant site x origin interaction in the dry season, with seedling survival in both sites dependent on their origin (Table 1, Fig 4e). Under natural control conditions in the dry site, wet origin species had a significantly lower survival than dry origin species (Table 2a). Wet origin species also had a lower survival in the dry than in the wet site (Table 2b). Survival in the exclosure followed the same pattern (Fig 4e), indicating that drought rather than pests led to the lower survival of wet origin species in the dry site. In contrast, dry origin species under control conditions showed no differences in survival between sites (Table 2b). Pest exclosure increased survival of wet origin species both in the dry and the wet site (Table 2c and 2d). For dry origin species, the exclosure effect was only marginally significant in the dry site (Table 2c). During the dry season, the triple interaction site x origin x treatment was not significant (Table 1). Light availability had no significant effects in the dry season (Table 1, Fig 4f), although the difference in light availability between the sites was highest during the dry season (Fig 2b), probably due to leaf shedding.

## Discussion

In contrast to our expectations, the overall establishment success (i.e. germination and one-year survival) did not reflect the distribution patterns of the species (Fig 3a). Under natural habitat conditions (i.e. exposed to pest pressure) seedlings had no home advantage in their respective home site, nor did their establishment success vary across sites (Fig 3a and Table 2a and 2b). Consistent with lower drought tolerance of wet origin species, dry season seedling survival in the dry site was significantly lower for wet than for dry origin species (Fig 4e, Table 2a). Although this did not result in an overall home advantage of dry forest species within the time-frame of our study (Fig 3), it may lead to the exclusion of wet forest species from dry forests in more intense dry seasons and over longer time frames.

Neither the pest pressure hypothesis, nor the light availability hypothesis were supported to be important in early life stages for excluding dry origin species from wet forests (Fig 3). However, growth patterns were consistent with a home advantage of wet origin species in the wet site (Fig 3c): Wet origin species grew significantly faster than dry origin species (Table 2a), and this effect was much more pronounced in the wet than in the dry site (significant site x origin interaction, Table 1). This home advantage of wet forest species was already visible one year after germination. It may accumulate over time, and lead to the eventual exclusion of dry origin species from wet sites. Below we discuss our results and their implications for factors and life stages shaping tree distributions across rainfall gradients in more detail.

### Exclusion of wet forest species from dry sites

Our results indicate that our focal wet forest species were less drought-tolerant than the dry forest species, and that drought limited their survival in the dry site during the dry season, as we had expected: Their dry season survival in the dry forest was much lower compared to dry forest species (Fig 4e, Table 2a), and compared to the wet forest site (Fig 4e, Table 2b). We can rule out that these effects were due to pest pressure, because seedlings in controls and exclosures followed the same pattern (Table 2c and 2d, Fig 4e), or that they were due to light, because light availability had no effect on seedling survival (Table 1, Fig 4f). Therefore, drought was directly responsible for reducing survival in the dry site. These results confirm previous studies in tropical forests worldwide which show that seedlings of wet forest species (or occurring in wet sites) are less drought-tolerant, i.e. more susceptible to drought, than seedlings of species occurring in dry forests exposed to a strong dry season [6, 8–10, 50].

Despite their lower drought tolerance, after one year wet forest species in the dry site did not perform poorer than dry forest species neither in terms of overall establishment nor growth (Fig 3a and 3c, Table 2a and 2b). Slightly, but non-significantly higher wet season survival in wet compared to dry origin species in the dry site (Fig 4c, Table 2a), may have counterbalanced their lower survival during the dry season, resulting in no overall difference in establishment success during our study period (Table 2a).

The strength of the dry season (i.e. the duration and the water deficit reached) varies considerably across years, and consequently, dry season seedling performance also varies [50]. Pronounced seedling mortality, especially of drought sensitive species, occurs predominantly in particularly dry years [50], while the dry season in our study period was well within the long-term average [45]. Rather than contradicting the physiological tolerance hypothesis, our results, together with the previous studies, thus underline the importance of strong and repeated dry seasons, such as those occurring during El Niño Southern Oscillation (ENSO) events, for exclusion of wet origin species from dry forests.

### Exclusion of dry forest species from wet sites

While the mechanisms underlying distribution limits of wet forest species to dry sites are quite well understood, the mechanisms excluding dry origin species from wet forests are not yet resolved. Contrary to our expectations, we found no indication of either high pest damage or low shade tolerance limiting the performance of dry origin species in the wet site.

#### Pest pressure hypothesis

Consistent with our hypotheses, pest exclosure through insecticide and fungicide treatment had a significant positive overall effect on establishment success, germination and survival (Table 1), indicating that these processes were limited by pests. Growth was not affected by pests in congruence with results from Eichhorn et al. [51], who argued that levels of herbivory were exceedingly high in the few studies which found negative effects of herbivore damage on growth.

However, contrary to our expectations, the performance of early life stages of dry origin species in the wet site was not limited by herbivores or pathogens, as shown by the lack of a positive effect of alleviating potential damage through pest exclosure (Table 2d). Furthermore, we did not find any indication of overall higher pest pressure in the wet site, which would have manifested itself in a significant site x treatment interaction with a higher treatment effect in the wet site (see Table 1). On the contrary, the effect of pest pressure was higher in the dry site for establishment success and germination, (see Table 1 and below). Thus, our results did not support the pest pressure hypothesis.

To our knowledge, so far only three studies have explicitly tested the pest pressure hypothesis. All three used transplant experiments with species of contrasting origins across tropical rainfall gradients [13, 14, 19]. Two studies in Panama [14, 19] found higher overall damage and higher pathogen damage in a wet aseasonal than in a dry seasonal forest, consistent with higher pest pressure but contrary to our results, while a study at the Malay-Thai peninsula found no evidence for higher pest pressure in an aseasonal compared to a seasonal forest [13]. None of these studies found significantly higher damage in dry than wet origin species indicative of lower defenses in the dry forest species, as required for the pest pressure hypothesis. Instead, the results of Spear et al. [19] suggest that the susceptibility of species, i.e. their likelihood to die after pathogen or herbivore damage, varies, with wet origin species being less susceptible. They proposed that higher susceptibility rather than lower defenses may limit the distribution of dry origin species in wet sites. However, if these processes are important for species distribution, an overall performance outcome consistent with the pest pressure hypothesis (i.e. stronger negative effects of pest on dry species performance) would still be expected, regardless if it is driven by defenses or susceptibility. Our results do not support the importance of differences in defenses nor susceptibility for germination or early seedling performance.

We expected higher pest limitation of dry forest species in wet forests, due to the combined effects of higher pest pressure in wetter forests and lower defenses of dry origin species (Fig 1). Instead, the positive effect of pest exclosure for dry origin species was consistently higher in the dry site compared to the wet site for all performance parameters (Figs 3 and 4, Table 2c and 2d), and for wet origin species the effect of pest exclosure was equally high in both sites (Figs 3 and 4, Table 2c and 2d). These results might hint towards a higher degree of specialization of the herbivore community in the wet than in the dry forest: If transplanting dry origin species to the wet forest introduced them to a specialized herbivore community with which they did not co-evolve, lower pest limitation compared to native wet forest species, as we observed, would be expected (compare enemy release hypothesis). On the other hand, if the herbivore community in the dry forest is more generalistic, wet origin species would be expected not to show higher release from pest pressure outside their home range, again consistent with our observation. If specialization of pests indeed increases across rainfall gradients, it would put the pest pressure gradient hypothesis into question, since dry forest species may escape their enemies and have an advantage in wet forests. While overall, specialization of insect herbivores and fungal pathogens is not as strong as originally thought [52, 53], we are not aware of any study comparing the degree of specialization of herbivore communities across rainfall gradients. Targeted studies analyzing specialization across rainfall gradients will be needed to evaluate this possibility.

In summary, our results do not support any of the patterns expected from the pest pressure hypotheses for early life stages, and—taken together with previous studies—decisive support for the pest pressure gradient hypothesis remains elusive.

#### Light availability hypothesis

Light responses in our experiment did not significantly differ between wet origin and dry origin species (no significant origin x light interaction, Table 1). We found no indication that dry origin species were more light-demanding than wet origin species, as expected from the light availability hypothesis. On the contrary, wet origin species even showed a trend to higher light requirements, indicated by the marginally significant trend to higher establishment success with increasing light than dry origin species (origin x light interaction, Table 1, Fig 3b). Previous studies similarly did not find support for higher light requirements in dry than wet forest species [8, 15, 21]. Additionally, although light availability was significantly higher in the dry than in the wet site (Fig 2), differences were small (see also [15]). Overall canopy openness showed only little variation with values between 1 and 7%. These values are typical within the understory of tropical forests [15, 25]. The small variation may contribute to the overall small effect of light on species performance observed in this study. Our results, together with previous studies, suggest that light does not play a significant role in shaping species distributions across tropical rainfall gradients.

#### Growth and the role of a drought-tolerance-growth trade-off

Wet forest species had a home advantage in terms of growth: in the wet forest growth rates of wet origin species were higher than of dry origin species (Table 2a). Through this growth difference, wet origin species may over time outperform and exclude dry origin species from wet forests. Previous studies in the area have also found lower growth rates in dry compared to wet forest species in independent species sets (only three species overlapping, [14, 36]). Similar patterns were also found in studies in the Malay-Thai peninsula, where widespread, dry distribution species had lower growth rates than aseasonal, wet distribution species [13, 54]. This suggests that lower seedling growth rates in dry than wet forest species are a general and widespread pattern.

We have discarded above that the lower growth rates of dry forest species were due to pest damage or light requirements. An alternative factor that may lead to this pattern is low nutrient availability in wet forests, and indeed high nutrient requirement of dry forest species have been suggested to exclude them from nutrient poor wet forests [2, 18, 36]. If dry origin species have higher nutrient requirements and wetter forests have lower nutrients, this could explain the reduced growth of dry origin species with increasing rainfall found in our study (Table 2b). However, dry and wet origin species do not differ in nutrient requirements in Central Panama [36], and relations between rainfall and nutrient availability are weak [4]. Differential nutrient requirements can therefore be ruled out as a cause for overall lower growth rates of dry forest species and for playing a major role in excluding dry origin species from wet forests, although they do influence distribution across nutrient gradients [4].

Instead, lower growth rates in dry forest species are consistent with a stress tolerance-growth trade-off, which has been hypothesized based on costs associated with adaptations to low resource availability which should lead to inherently lower growth rates, even under optimum conditions, in stress-tolerant species [20, 27]. There is ample evidence for a stress tolerance-growth trade-off based on shade (e.g. [55]). Also, several traits promoting tolerance to drought are traded-off against growth rates [28, 29]. Nevertheless, although often implied, direct empirical evidence for a whole-plant drought tolerance-growth trade-off remains surprisingly scarce. Support for a trade-off between drought survival and maximum growth rates or shoot growth rate across species was found e.g. by O’Brien et al. [29], Polley et al. [31] and Wikberg et al. [32], in tropical tree seedlings, tropical and subtropical woody legumes, and in willows, respectively. Consistently, in our study there was a marginally significant negative relation between dry season survival on the dry site and maximum growth rates (assessed as the upper 95 percentile of growth on the wet site, GLMER: p = 0.07, based on data for the 16 species with more than 3 survivors). However, the only rigorous experimental study that explicitly tested for this trade-off, which was conducted in eight desert grasses, did not support it [30].

Species with dry distribution have been experimentally shown to be more tolerant to drought stress than species with wet distribution [8, 9, 54], and higher drought-tolerance in dry origin species is consistent with the data from our study (see above). Inherently lower growth rates of dry compared to wet origin species found in this (Table 2a) and other studies [14, 36, 54] thus provide additional indirect support for a stress tolerance-growth trade-off with respect to drought. This trade-off may underlie the exclusion of dry forest species from wet sites and be fundamental in shaping species distributions along rainfall gradients.

### The role of early life stages for species distributions

In this study, we focused on the initial life stages of germination and early seedling establishment, since these stages are considered the most vulnerable in the face of biotic and abiotic stressors [33, 34] and may thus be critical in shaping species distribution patterns across tropical rainfall gradients. However, germination (i.e. radicle emergence) patterns did not reflect the occurrence patterns of the species (Fig 4a, Table 2a and 2b), indicating that species partitioning along the rainfall gradient did not occur at this stage. We found support that differential dry season survival (in dry sites) and differential growth (in wet sites) during early life stages contribute to shaping tree distribution patterns across tropical rainfall gradients (see above). However, effects were weak and not sufficient to lead within the initial year to a clear home advantage of the species on the dry or wet side, respectively. Across a topographic moisture gradient, processes within one year after emergence were also insufficient to explain habitat preferences of adult plants [33]. This strongly suggests that longer time spans reaching into later life stages, and repeated and pronounced dry seasons are important for filtering tree distribution patterns.

The importance of later life stages and longer time periods for shaping distribution patterns is supported by local scale studies: If habitat associations of adults are shaped by failure to germinate or to establish, older juveniles and adults should exhibit the same habitat associations. However, most species have different associations at seedling and late life stages [56].

## Conclusions

We found two processes that may lead to the differential distribution patterns of dry and wet origin species after longer time periods and at later life stages. We showed that drought limits the survival of wet origin species in dry forests, which supports the physiological tolerance hypothesis. Dry origin species had lower growth rates than wet origin species, especially in the wet forest site, consistent with a drought-tolerance- growth trade-off. Our results support that repeated and intense dry season drought limits performance and consequent distribution of wet origin species in dry forests, and suggest that dry origin species are outperformed in wet forests due to inherently lower growth rates, based on a drought-tolerance- growth trade-off.

Although pest pressure had a strong overall influence on species establishment success, we found no support for the hypothesis that high pest pressure excludes dry origin species from wet forests (pest pressure gradient hypothesis). We also found no evidence for the hypothesis that dry origin species have higher light requirements than wet origin species, and are thus excluded from wetter forests with darker understory (light gradient hypothesis).

Our results underline that changes in water availability due to climate change will have direct consequences on species regeneration and distributions along rainfall gradients, while indirect effects of pest pressure and light availability play a subordinate role.

## Supporting Information

S1 Dataset Dataset contains raw data including plot designation, species names, individual performance parameters (establishment success, growth, germination, dry season survival, wet season survival), and environmental data in different seasons (canopy openness and gravimetric soil moisture). (XLS)

S1 Fig Performance parameters of the 26 focal species analyzed. Probability of establishment success (A), growth (B), probability of germination (C), probability of wet season survival (D) and probability of dry season survival (E), sorted by species’ origin (dry: red, wet: blue) and average establishment success. Data are averages and standard errors. Species effects on all performance parameters were highly significant (GLMM for probability of establishment, germination, total survival, survival wet and dry season and LMM for growth: p < 0.001). For full species names see S1 Table.
(PDF)

S1 Table Focal species, including their classification into dry or wet origin. (PDF)

S2 Table Correlations of site and abiotic factors. (PDF)

S3 Table Detailed summary table of the Models (GLMM and LMM). (PDF)
