# Supplementary material for: Effects of Drought, Pest Pressure and Light Availability on Seedling Establishment and Growth: Their Role for Distribution of Tree Species across a Tropical Rainfall Gradient
Source: PLoS One. 2015 Nov 30;10(11):e0143955. doi: 10.1371/journal.pone.0143955 (PMC4664389; doi:10.1371/journal.pone.0143955)
Supplement: S1 Fig — Probability of establishment success (A), growth (B), probability of germination (C), probability of wet season survival (D) and probability of dry season survival (E), sorted by species’ origin (dry: red, wet: blue) and average establishment success. Data are averages and standard errors. Species effects on all performance parameters were highly significant (GLMM for probability of establishment, germination, total survival, survival wet and dry season and LMM for growth: p < 0.001). For full species names see S1 Table. (PDF) [file pone.0143955.s002.pdf]

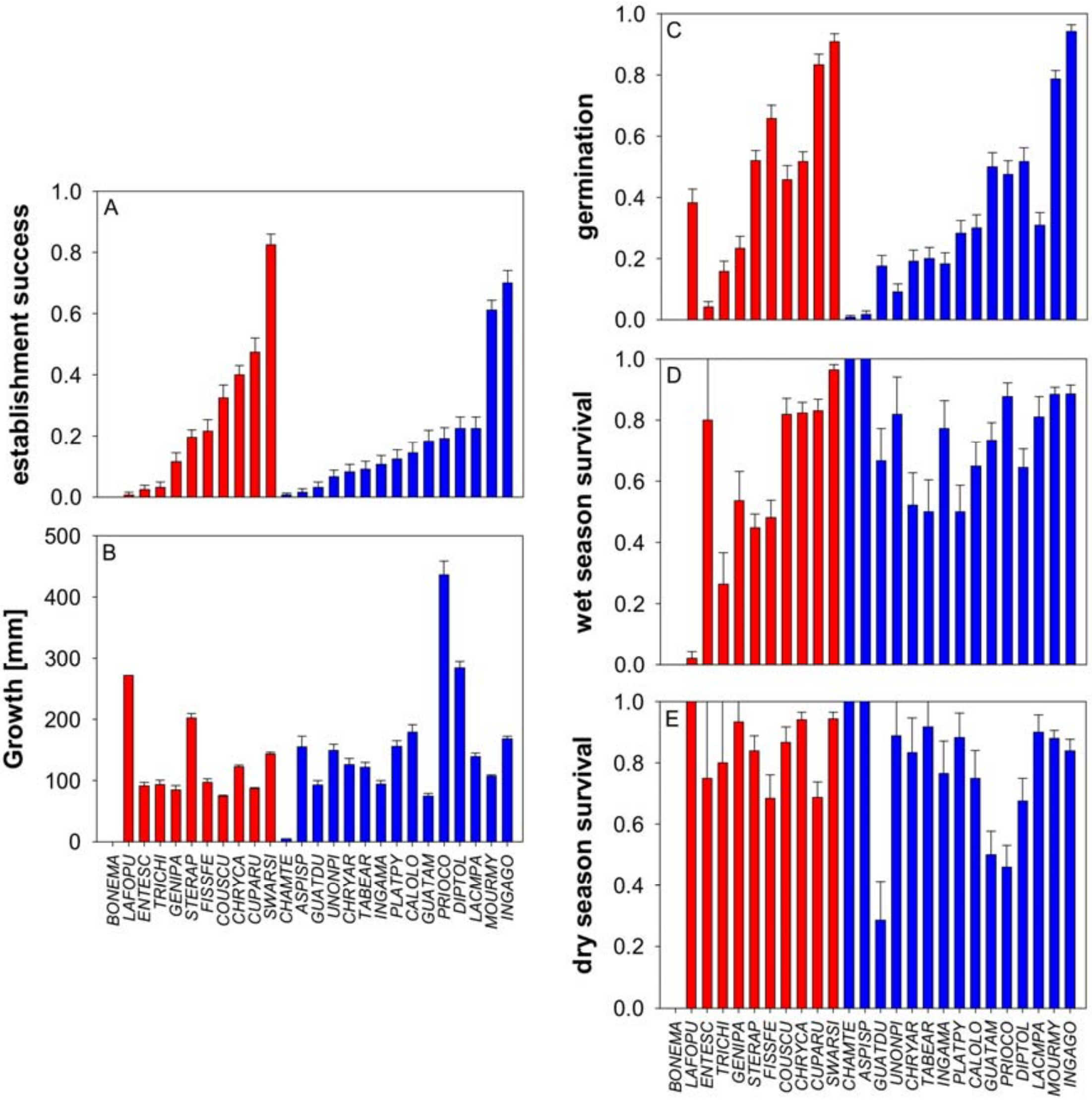

S1 Fig. Performance parameters of the 26 focal species analyzed. Probability of establishment success (A), growth (B), probability of germination (C), probability of wet season survival (D) and probability of dry season survival (E), sorted by species' origin (dry: red, wet: blue) and average establishment success. Data are averages and standard errors. Species effects on all performance parameters were highly significant (GLMM for probability of establishment, germination, total survival, survival wet and dry season and LMM for growth: $p < 0.001$). For full species names see Table S1.
