# Supplementary material for: Effects of Drought, Pest Pressure and Light Availability on Seedling Establishment and Growth: Their Role for Distribution of Tree Species across a Tropical Rainfall Gradient
Source: PLoS One. 2015 Nov 30;10(11):e0143955. doi: 10.1371/journal.pone.0143955 (PMC4664389; doi:10.1371/journal.pone.0143955)
Supplement: S1 Table — (PDF) [file pone.0143955.s003.pdf]

S1 Table. Focal species, including their classification into dry or wet origin.

species	abbreviation	family (-ceae)	origin	criteria for classification
Aspidosperma spruceanum Benth. ex Müll. Arg.	ASPISP	Apocyna	wet	1
Bonellia macrocarpa (Cav.) B. Ståhl & Källersjö	BONEMA	Primula	dry	3
Calophyllum longifolium Willd.	CALOLO	Calophylla	wet	1
Chamaedorea tepejilote Liebm.	CHAMTE	Areca	wet	1
Chrysophyllum argenteum Jacq.	CHRYAR	Sapota	wet	1,2
Chrysophyllum cainito L.	CHRYCA	Sapota	dry	1,2
Coussarea curvigemmia Dwyer	COUSCU	Rubia	dry	1
Cupania rufescens Triana & Planch.	CUPARU	Sapinda	dry	1
Dipteryx oleifera Benth.	DIPTOL	Faba	wet	1
Enterolobium schomburgkii (Benth.) Benth.	ENTESC	Faba	dry	1
Fissicalyx fendleri Benth.	FISSFE	Faba	dry	1
Genipa americana L.	GENIAM	Rubia	dry	1,2
Guatteria amplifolia Triana & Planch.	GUATAM	Annona	wet	1
Guatteria dumetorum R.E. Fr.	GUATDU	Annona	wet	1
Inga goldmanii Pittier	INGAGO	Faba	wet	1,2
Inga marginata Willd.	INGAMA	Faba	wet	1
Lacmellea panamensis (Woodson) Markgr.	LACMPA	Apocyna	wet	1
Lafoensia punicifolia DC.	LAFOPU	Lythra	dry	1
Mouriri myrtilloides (Sw.) Poir.	MOURMY	Melastomata	wet	1
Platymiscium pinnatum (Jacq.) Dugand	PLATPI	Faba	wet	1
Prioria copaifera Griseb.	PRIOCO	Faba	wet	1
Sterculia apetala (Jacq.) H. Karst.	STERAP	Malva	dry	1
Swartzia simplex (Sw.) Spreng.	SWARSI	Faba	dry	1
Tabernaemontana arborea Rose	TABEAR	Apocyna	wet	1
Trichilia hirta L.	TRICHI	Melia	dry	1
Unonopsis pittieri Saff.	UNONPI	Annona	wet	3

Criteria used:

1: occurrence maps according to CTFS [42], available at

http://ctfs.arnarb.harvard.edu/webatlas/maintreeatlas.php

2: abundance data according to CTFS [42], available at

http://ctfs.arnarb.harvard.edu/webatlas/datasets/

3: description of the species according to CTFS [42]
