# Supplementary material for: Effects of Drought, Pest Pressure and Light Availability on Seedling Establishment and Growth: Their Role for Distribution of Tree Species across a Tropical Rainfall Gradient
Source: PLoS One. 2015 Nov 30;10(11):e0143955. doi: 10.1371/journal.pone.0143955 (PMC4664389; doi:10.1371/journal.pone.0143955)
Supplement: S2 Table — (PDF) [file pone.0143955.s004.pdf]

S2 Table. Correlations of site and abiotic factors.

	Site	Light dry	Light wet	Light mean	Moisture dry 1	Moisture wet	Moisture dry 2
Light dry	-0.64						
Light wet	-0.36	0.47					
Light mean	-0.63	0.92	0.74				
Moisture dry 1	0.87	-0.66	-0.25	-0.60			
Moisture wet	0.82	-0.59	-0.14	-0.50	0.89		
Moisture dry 2	0.92	-0.65	-0.30	-0.58	0.79	0.78	
Moisture mean	0.85	-0.61	-0.16	-0.52	0.90	0.99	0.82

Light dry, wet and mean: % canopy openness during the dry season, the wet season, and the mean, respectively. Moisture dry 1, wet, dry 2 and mean: % gravimetric soil moisture during the transition between first dry season and wet season, during the wet season, during the second dry season, and mean soil moisture. Values are Spearman rank correlation factors (r).
