# Supplementary material for: Effects of Drought, Pest Pressure and Light Availability on Seedling Establishment and Growth: Their Role for Distribution of Tree Species across a Tropical Rainfall Gradient
Source: PLoS One. 2015 Nov 30;10(11):e0143955. doi: 10.1371/journal.pone.0143955 (PMC4664389; doi:10.1371/journal.pone.0143955)
Supplement: S3 Table — (PDF) [file pone.0143955.s005.pdf]

S3 Table. Summary table of the Models (GLMM and LMM).

		Establishment success	Growth	Germination	Survival wet season	Survival dry season
intercept	Estimate	-2.187	4.851	-0.904	-0.141	1.457
	z (t) value	-3.224	168.4	-1.545	-0.241	3.687
	p value	**0.001**	**<0.001**	0.122	0.81	**<0.001**
site	Estimate	0.183	-0.199	0.340	-0.286	0.053
	z (t) value	0.715	-4.716	1.890	-0.949	0.175
	p value	0.474	**<0.001**	*0.059*	0.343	0.861
origin	Estimate	-1.110	0.096	-0.334	0.544	-1.112
	z (t) value	-1.283	2.31	-0.435	0.863	-2.291
	p value	0.199	**0.021**	0.664	0.388	**0.022**
treatment	Estimate	0.841	0.039	0.575	0.711	0.751
	z (t) value	4.264	1.306	3.348	2.431	4.167
	p value	**<0.001**	0.192	**<0.001**	**0.015**	**<0.001**
canopy openness	Estimate	-0.031	-0.031	-0.015	0.284	0.049
	z (t) value	-0.212	-1.213	-0.236	1.963	0.401
	p value	0.832	0.226	0.813	**0.049**	0.689
site x origin	Estimate	0.078	0.166	-0.133	-0.319	0.863
	z (t) value	0.242	2.816	-0.561	-0.824	2.312
	p value	0.809	**0.005**	0.574	0.41	**0.021**
site x treatment	Estimate	-0.880	-0.024	-0.737	-0.57	0.278
	z (t) value	-3.135	-0.399	-3.042	-1.451	0.764
	p value	**0.002**	0.69	**0.002**	0.147	0.445
origin x treatment	Estimate	-0.199	0.063	-0.302	0.322	0.445
	z (t) value	-0.721	1.052	-1.271	0.75	1.208
	p value	0.471	0.293	0.204	0.453	0.227
origin x canopy openness	Estimate	0.338	-0.018	0.158	0.278	0.324
	z (t) value	1.931	-0.343	1.477	1.137	1.491
	p value	*0.054*	0.732	0.140	0.256	0.136
site x origin x treatment	Estimate	1.221	-0.052	0.623	0.968	1.095
	z (t) value	3.145	-0.436	1.863	1.689	1.483
	p value	**0.002**	0.663	*0.062*	*0.091*	0.138

Estimate (regression coefficient), z- value and p-value of the explanatory variables site, origin, treatment and canopy openness are given for the response variables establishment success, germination, overall survival, wet and dry season survival. For growth, t- value is given instead of z- value.

Bold p-values show significant effects at the 0.05 level, italic p-values close-significant trends at the 0.1 level.
